# Early Alterations of Corneal Subbasal Plexus in Uncomplicated Type 1 Diabetes Patients

**DOI:** 10.1155/2019/9818217

**Published:** 2019-07-02

**Authors:** Domenico Schiano Lomoriello, Irene Abicca, Mariacristina Parravano, Daniela Giannini, Benedetta Russo, Simona Frontoni, Fabiana Picconi

**Affiliations:** ^1^IRCCS–Fondazione Bietti, Rome, Italy; ^2^Unit of Endocrinology, Diabetes and Metabolism, S. Giovanni Calibita Fatebenefratelli Hospital, Rome, Italy; ^3^Unit of Endocrinology, Diabetes and Metabolism, S. Giovanni Calibita Fatebenefratelli Hospital, Department of Systems Medicine, University of Rome Tor Vergata, Rome, Italy

## Abstract

**Purpose:**

The purpose of our study is to describe the in vivo corneal confocal microscopy characteristics of subbasal nerve plexus in a highly selected population of patients affected by type 1 diabetes mellitus (T1DM) without any microvascular diabetes complications.

**Methods:**

We included 19 T1DM patients without diabetic peripheral neuropathy, diabetic autonomic neuropathy, diabetic retinopathy, and microalbuminuria. All patients underwent in vivo corneal confocal microscopy and blood analysis to determine subbasal nerve plexus parameters and their correlation with clinical data. We compared the results with 19 healthy controls.

**Results:**

The T1DM group showed a significant decrease of the nerve fiber length (*P*=0.032), the nerve fiber length density (*P*=0.034), the number of fibers (*P*=0.005), and the number of branchings (*P*=0.028), compared to healthy subjects. The nerve fiber length, nerve fiber length density, and number of fibers were directly related to the age at onset of diabetes and inversely to the duration of DM. BMI (body mass index) was highly related to the nerve fiber length (*r* = −0.6, *P*=0.007), to the nerve fiber length density (*r* = −0.6, *P*=0.007), and to the number of fibers (*r* = −0.587, *P*=0.008). No significant correlations were found between the corneal parameters and HbA1c.

**Conclusions:**

Early subclinical fiber corneal variation could be easily detected using in vivo corneal confocal microscopy, even in type 1 diabetes without any microvascular diabetes complications, including diabetic peripheral neuropathy, diabetic autonomic neuropathy, diabetic retinopathy, and microalbuminuria.

## 1. Introduction

Diabetic neuropathy (DN) is an important complication of diabetes mellitus (DM). An early diagnosis of DN has an increasing importance in diabetes management, so the literature is focused on identifying predictive factors for the onset of DN [1]. Alterations of morphological and structural parameters of corneal nerves in patients affected by DM have been already described in the literature [2]. Recently, corneal neuropathy has been suggested as a predictive sign of peripheral neuropathy. In particular, structural changes of the subbasal plexus (SBP) have been reported [3].

In vivo corneal confocal microscopy (IVCCM) is a noninvasive, rapid, and repeatable technique used to obtain in vivo images of the corneal structure, from the endothelium to the epithelium. Therefore, it is considered a diagnostically valid examination for the evaluation of systemic neuropathic processes. With IVCCM, a reduction of fiber density and an increase of fiber tortuosity, related to diabetic peripheral neuropathy (DPN) severity, has been observed in corneal subbasal nerve fibers in patients with DPN [3–5]. Moreover, it has been suggested that chronic hyperglycemia could induce a mitochondrial dysfunction, through an increased oxidative metabolism [3, 6]. Accordingly, several studies based on IVCCM exams reported mitochondria larger in shape and less metabolically active. Those accumulations of mitochondria and glycogen particles appear as local axon enlargement called beadings [7, 8].

The aim of our study is to describe the characteristics of corneal SBP in a highly selected population of subjects affected by type 1 diabetes (T1DM), without microvascular diabetes complications, including DPN, diabetic autonomic neuropathy (DAN), diabetic retinopathy (DR), and microalbuminuria.

## 2. Materials and Methods

### 2.1. Study Population

We have screened patients referring to the Unit of Endocrinology, Diabetes and Metabolism, Department of Systems Medicine in S. Giovanni Calibita Fatebenefratelli Hospital, University of Rome Tor Vergata, Rome, Italy, from March 1, 2017, to March 30, 2018. All patients enrolled were aged >18 years, and they were affected by T1DM, according to American Diabetes Association (ADA) criteria [9]. Exclusion criteria were as follows:

1. Symptomatic peripheral diabetic polyneuropathy even without positive sensory symptoms such as pain, burning, paraesthesia, or prickling
2. A Michigan Diabetes Neuropathy Instruments [10] total score equal to or greater than 2 points
3. DAN evaluated by Ewing battery [11]
4. History of possible confounding diseases (inﬂammatory diseases, alcohol abuse, vitamin deficiency, malignancy treated with chemotherapy agents, recent history of heart or respiratory failure, chronic liver or renal failure central nervous system diseases, entrapment mononeuropathies, and cervical or lumbosacral radiculopathies)
5. Microalbuminuria (urinary albumin/creatinine ratio >30 mg/g)

All patients underwent a general medical examination and anthropometric parameters. After an overnight fast, blood and urine samples were obtained for the determination of laboratory measurements. We performed blood tests to measure TGL, CT, HDL, LDL, and creatinine in all diabetes mellitus type 1 patients in order to describe the metabolic characteristics of the population and to rule out the confounding effect of high lipid values or renal failure on SBP parameters. Regarding healthy subjects, we performed an oral glucose tolerance test in order to exclude diabetes and impaired glucose tolerance. We, also, excluded subjects with dyslipidemia, chronic renal failure, and hypertension based on the medical history. A complete ophthalmic examination was carried out in all subjects recruited for the study. Ocular exclusion criteria were the diagnosis of diabetic retinopathy (DR), contact lenses wearing, history of refractive, glaucoma or retinal surgery, ocular medications, with the exception of artificial tears, cataract surgery within the last 6 months, and eye inflammation. All research procedures described in this work adhered to the tenets of the Declaration of Helsinki. All subjects recruited allowed written informed consent after a full explanation of the procedure.

### 2.2. Laboratory Measurements

Blood and urinary samples were analysed as described in a previous work of our group [12].

HbA1c was quantified by high-performance liquid chromatography (VARIANT 2; BioRad Laboratories, Munich, Germany), with intra- and interassay CV of 0.46–0.77 and 0.69–0.91%, respectively. Plasma total cholesterol, high-density lipoprotein cholesterol (HDL-C), and low-density lipoprotein cholesterol (LDL-C) were analysed with a colorimetric enzymatic method (CHOD-PAP; Roche Diagnostics). The intraassay CV was 1%, and the interassay CV was 2.7%. The sensitivity of the method was 0.08 mmol/L. Plasma triglycerides were analysed with a colorimetric enzymatic method (GPO-PAP; Roche Diagnostics). The intraassay CV was 1.5%, and the interassay CV was 2.4%. The sensitivity of the method was 0.05 mmol/L. Urinary albumin was determined by the Tina-quant immunoturbidimetric assay (Cobas; Roche Diagnostic, Indianapolis, IN) and urinary creatinine by the enzymatic colorimetric test (Beckmann Coulter, California, USA).

### 2.3. In Vivo Corneal Confocal Microscopy (IVCCM)

IVCCM (Confoscan 4; Nidek Technologies, Gamagori, Japan) was performed bilaterally on the central cornea of all patients at the anterior segment unit of IRCSS Fondazione Bietti, Rome, Italy.

A total of 19 healthy patients matched by sex and age were included as control.

After the application of one drop of topical aneasthetic, 0.4% oxybuprocaine chlorohydrate (Novesina, Novartis Farma, Varese, Italy), a transparent and sterile viscous gel (dexpantenol 5%) was applied to the tip of the lens. This eliminates the optical interfaces with different refractive indices, keeping constant the refractive index, and allows to maintain the desired focal distance. Furthermore, the interposition of the gel allowed a no-contact examination with invasiveness. The *z*-ring was used in all cases. The standard dimension of each image was 340 × 255 *μ*m, with an optical section thickness of 5.50 *μ*m. The overall examination took 2 to 3 minutes. Nobody among patients complained corneal symptoms or visual complications after the examination.

### 2.4. Corneal Subbasal Nerve Plexus Analysis

The images have been selected from the layer immediately at, or posterior to, the basal epithelial layer and anterior to Bowman's layer. For each patient, the best focused frame of the SBP was chosen.

**Figure 1:**
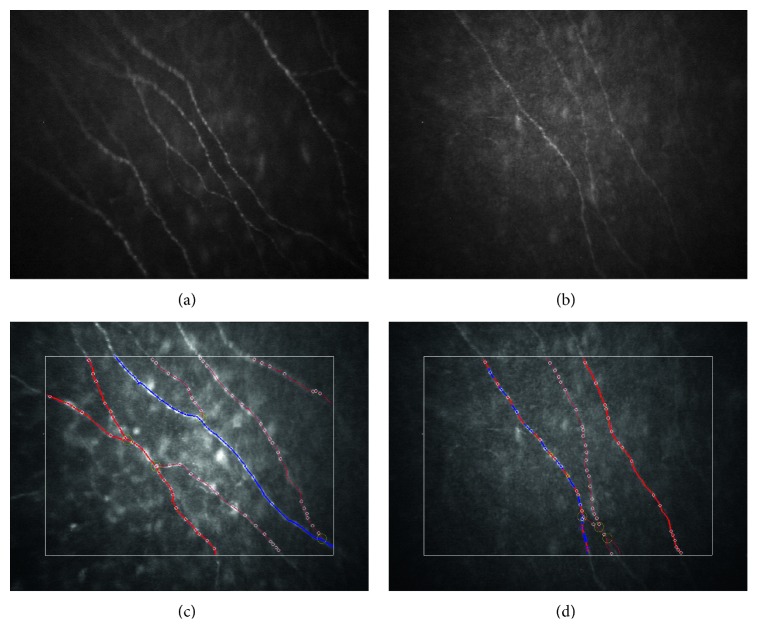
Representative IVCCM images of (a) a healthy control; (b) a T1DM patient with no DPN; (c) corneal nerve fiber image analysis using CS4 Nerves Tracking software of a healthy control; (d) corneal nerve fiber image analysis using CS4 Nerves Tracking software of a T1DM patient.

The analysis of corneal nerve fibers was performed later using CS4 Nerves Tracking Tool CS4 software v1.3.0 and manual edit (Figure 1).

All examinations were obtained by the same experienced operator (DSL), who selected the best focused image for each patient. A second, masked, experienced operator (IA) performed the analysis of frames. After automated identification of fibers, two operators (DSL and IA), who were masked to group assignment, reviewed each area and manually corrected any error.

The corneal SBP parameters analysed were 7 and are as follows:

1. Nerve fiber length, the total length of all fibers and branches/frame (*µ*m/frame)
2. Nerve fiber length density, the total length of the nerve fibers in *µ*m/mm^2^
3. Number of fibers, the total number of nerve fibers, including main nerves and branches
4. Number of branchings, points where nerve branches arise from main nerve
5. Number of beadings, the total number of well-defined hyperreflective points in all identified main nerves (trunks, long fibers that crossed the borders of the area of analysis in one image)
6. Beadings density, the total number of nerve beadings divided by the total length of nerve trunks in millimeter (beadings/mm)
7. Nerve fiber tortuosity using Nidek Nerve index, a unitless measure which represents the degree of twistedness of a curved structure

## 3. Statistical Analysis

Statistical evaluation was considered using SPSS (IBM SPSS Statistics 25). All results were expressed as the mean ± standard deviations. The normal data distribution was tested by using the one-sample Kolmogorov–Smirnov test. In order to compare differences in parameters between the diabetic patient group and healthy subjects, the independent-sample *t* test and the Mann–Whitney test were used as appropriate. To study the relationship between parameters, the Pearson correlation coefficient was computed. In all analyses, *P* < 0.05 was considered to be statistically significant.

## 4. Results

A total of 19 patients affected by T1DM were included (10 females and 9 males) and compared to a healthy control group of 19 patients (10 females and 9 males).

The clinical and demographic characteristics of the T1DM group are described in Table 1. Both groups were comparable by age (T1DM 37.42 ± 8.99 versus control 40.31 ± 11.15, *P*=0.384).

All participants underwent corneal SBP analysis. The nerve fiber length (T1DM group (866.45 ± 432.04) versus control group (1186.20 ± 450.02), *P*=0.032), the nerve fiber length density (T1DM group (9808.96 ± 4808.96) versus control group (13357.22 ± 5056.19), *P*=0.034), the number of fibers (T1DM group (4.68 ± 2.11) versus control group (7.16 ± 2.87), *P*=0.005), and the number of branchings (T1DM group (1.89 ± 1.56) versus control group (3.26 ± 1.99), *P*=0.028) were significantly lower in the T1DM group compared to those in the healthy subjects, while the number and density of beadings and nerve fiber tortuosity did not differ between the two groups (Table 2). In T1DM group, the age at onset of diabetes was directly related to the nerve fiber length (*r* = 0.535, *P*=0.018), the nerve fiber length density (*r* = 0.524, *P*=0.02), and the number of fibers (*r* = 0.444, *P*=0.05). The same SBP parameters were inversely related to the duration of DM (nerve fiber length: *r* = −0.657, *P*=0.002; nerve fiber length density: *r* = −0.666, *P*=0.002; number of fibers: *r* = −0.610, *P* = 0.006). None of nerve fiber parameters was related to the age of the patients at the time of the examinations (Table 3). No significant correlations were found between the corneal parameters and HbA1c. BMI of the T1DM group was highly related to the nerve fiber length (*r*=−0.6, *P*=0.007), the nerve fiber length density (*r*=−0.6, *P*=0.007), and the number of fibers (*r*=−0.587, *P*=0.008) (Table 4).

We also compared T1DM and healthy groups, according to the sex. Females were comparable by age, and all corneal SBP parameters did not statistically differ between healthy and T1DM subjects. Analysing the SBP data in males, instead, the nerve fiber length (T1DM group (697.96 ± 101.48) versus control group (1415.42 ± 132.39), *P*=0.001), the nerve fiber length density (T1DM group (7978.80 ± 1158.24) versus control group (15932.77 ± 1489.73), *P*=0.001), the number of fibers (T1DM group (4.11 ± 0.75) versus control group (8.78 ± 0.83), *P*=0.001), the number of branchings (T1DM group (1.78 ± 0.57) versus control group (3.89 ± 0.73), *P*=0.026), and the number of beadings (T1DM group (16.67 ± 1.21) versus control group (21.11 ± 1.36), *P*=0.029) were found to be significantly lower in T1DM males, compared to healthy males. Age, beading density, and corneal nerve tortuosity did not differ between diseased and healthy males (Table 5). Dividing the patients within the diabetic group by sex, we observed that the nerve fiber length (male T1DM (697.96 ± 101.48) versus female T1DM (1018.09 ± 153.95); *P*=0.05) and the nerve fiber length density (male T1DM (7978.80 ± 1158.24) versus female T1DM (11456.11 ± 1732.28); *P*=0.05) were statistically lower in males compared to females. However, this difference was caused by the lower age at onset of diabetes in males compared to females (male T1DM (19.55 ± 2.52) versus female T1DM (29.8 ± 3.19), *P*=0.045), as we found repeating the statistical analysis on studentized residuals after correction for age at onset (fiber length *P*=0.106; fiber length *P*=0.097). BMI and blood parameters did not differ between female and male in the T1DM group (Table 6).

In healthy controls, none of corneal SBP parameters was sex-related. On the other hand, in T1DM males, we found an inverse relation between the duration of DM and two corneal parameters, the nerve fiber length (*r* = −0.690, *P*=0.04), and the nerve fiber length density (*r* = −0.718, *P*=0.029). BMI had an indirect correlation with the nerve fiber length (*r* = −0.856, *P*=0.003), the nerve fiber length density (*r* = −0.855, *P*=0.002), and the number of fibers (*r* = −0.774, *P*=0.014). In the female subgroup of T1DM, we did not found any correlation with clinical age, age at onset, and DM duration (Table 7).

## 5. Discussion

The utility of IVCCM to define the corneal SBP in diabetic patients with or without DPN has already been reported [4, 13]. The concept of corneal neuropathy with corneal fiber damages in diabetes was introduced by the Malik group [14], describing a significant reduction of corneal nerve fiber density, length, and branch density in diabetic patients with DPN compared to healthy controls. The aim of our study was to investigate the characteristics of corneal SBP in adult T1DM without DPN and any other ocular signs or symptoms, including DR, and to correlate them with clinical and anthropomorphic data. We excluded patients affected by DR from our population, because a relationship between the decrease of number of corneal fiber nerve and severity of DR has already been described [15, 16]. Furthermore, diabetic neuropathy and retinal neurodegeneration could anticipate a clinical evident retinopathy [12, 16].

In our study, we observed that the nerve fibers length, the nerve fibers density, the number of fibers, and the number of branchings were statistically lower in patients affected by T1DM compared to nondiabetic controls. These differences were observed in our group of highly selected diabetic subjects without any microvascular complications. Moreover, the good glycemic and metabolic control and the lack of comorbidities allowed to carry out these evaluations without confounding factors.

Our results were aligned to other studies in the literature. Ishibashi's group described that, in corneal SBP, the nerve fiber length, the nerve fiber density, and the branch density were lower in patients affected by T2DM without DPN, compared with those of healthy controls [6].

Edwards et al., comparing diabetic patients with and without DPN, demonstrated the nerve fiber length and the nerve branch density reduction in patients affected by DPN [17]. In particular, corneal nerve fiber length could be considered predictive of DPN [18, 19].

Other studies already described SBP alteration in T1DM without DR compared to that in healthy controls [15, 16]. They reported a reduction of the nerve fiber density and the nerve fiber length. Our study, compared to the previous study of Petropoulos and Burdova, examined also the metabolic activity of the fiber, expressed as number and density of beadings [8].

Regarding beadings, we did not detect any difference between our diabetic patients and controls. Our results about beadings were in contrast with the studies of Ishibashi's group, where they described a lower beading frequency in T1DM without DPN, compared to controls and a consequently reduction of the number of beadings and alteration of their size [6, 20]. They hypothesized that these alterations could be caused by changes in the distribution of mitochondria, which became detectable before the onset of DPN. We supposed that in our population of adult T1DM without DPN, the good glycemic and metabolic control could justify the absence of this difference in beading parameters of diabetics, compared to controls. However, this difference could be also due to the different methodologies. Indeed, both Ishibashi and Tavakoly used a manual method to count the beadings and numbered the beadings for 0.1 mm of fiber, while in our study, we performed an automatic count and revised manually [2, 21]. Moreover, Ishibashi did not specify whether T1DM patients were affected by DR, which could be associated with an early degeneration of corneal fibers, with a possible variation of beadings too.

In our population, the tortuosity index did not differ from controls as well. This is opposite to the previous reported data by Kallinikos et al., who found that the fiber tortuosity index seemed to be related to the degenerative mechanism and the regenerative response of nerve fibers in diabetes [22]. Moreover, the difference in this result could be justified by the fact that our population of diabetics did not have DPN, unlike the Kallinikos study group.

In our study, age at onset was directly associated to the nerve fibers length, the nerve fibers density, and the number of fibers. These SBP parameters were instead inversely related to DM duration. These results were in agreement with the current literature [16, 22]. None of the corneal parameters were related to the age of patients at the time of exams. It has been already described that fiber nerve number and density and also the number of beadings did not statistically reduce with age in young adults [16, 23, 24].

As for the clinical data, the nerve fibers length, the nerve fibers density, and the number of fibers were inversely related to BMI. In our study, we also confirmed the lack of correlation between HbA1c and corneal parameters, as already reported in the literature [25, 26].

We had divided our study population according to the sex. In female, there was no difference between the SBP parameters of T1DM and those of healthy controls. On the other hand, T1DM males showed a reduction in corneal nerve fiber length, corneal nerve fiber length density, the number of fibers, the number of branchings, and the number of beadings compared to healthy males. Therefore, we studied the differences in corneal nerves parameters in T1DM divided by sex and we found that fiber length and fiber length density were lower in diseased males than in females ones. However, analysing our data, it was found that these differences were not sex-related, but rather related to the age of onset of diabetes, which was earlier in males than in females. These results underline the importance of the age of onset on corneal parameters alterations.

The main limitation of the assessment of our results was the small number of the examined patients, slightly due to the strict inclusion criteria we assumed. Indeed, we excluded all adult diabetics with any microvascular complication, including DPN, DAN, DR, and microalbuminuria. Nevertheless, our study demonstrates the presence of corneal SBP alterations even in a highly selected subgroup of diabetics. A longitudinal study carrying out an IVCCM in a larger group of diabetic patients before the onset of disease-related complications would be further investigated.

## 6. Conclusions

An alteration of corneal subbasal plexus is already present in subjects affected by T1DM highly selected, without microvascular complications and comorbidities, and in good glycemic and metabolic controls. IVCCM confirms to be a noninvasive and helpful tool in the diagnosis of early diabetic alterations.

## Figures and Tables

**Table 1:** Demographic, metabolic, and anthropometric characteristics of the study population.

	T1DM (*n* = 19)
Age (mean ± SD) (years)	37.42 ± 8.99
Sex (male/female)	10/9
Age at onset of DM (years)	24.94 ± 10.18
Duration of DM (years)	12.47 ± 8.29
BMI (Kg/m^2^)	25.03 ± 4.63
HBA1c (%)	7.62 ± 0.84
TG (mg/dl)	77 ± 30.45
TC (mg/dl)	180.95 ± 26.94
HDL-C (mg/dl)	66.37 ± 15.96
LDL-C (mg/dl)	99.71 ± 24.97
Creatinine (mg/dl)	0.77 ± 0.12
Microalbuminuria/creatininuria (mg/g)	8.62 ± 8.13

BMI, body mass index; HbA1c, glycosylated hemoglobin; TG, triglycerides; TC, total cholesterol; LDL-C, low-density lipoprotein-cholesterol; HDL-C, high-density lipoprotein-cholesterol.

**Table 2:** Summary of corneal nerves morphological parameters in study population (T1DM versus healthy control).

Corneal nerves parameters	Healthy control (*n* = 19)	T1DM (*n* = 19)	*P* value
Nerve fiber length (*µ*m/frame)	1186.20 ± 450.02	866.45 ± 432.04	0.032^*∗*^
Nerve fiber length density (*µ*m/mm^2^)	13357.22 ± 5056.19	9808.96 ± 4853.05	0.034^*∗*^
Number of fibers (no./mm^2^)	7.16 ± 2.87	4.68 ± 2.11	0.005^*∗*^
Number of branchings (no.)	3.26 ± 1.99	1.89 ± 1.56	0.028^*∗*^
Number of beadings (no.)	19.68 ± 3.54	16.21 ± 5.12	0.056
Beadings density (no./mm)	71.37 ± 10.30	65.62 ± 21.85	0.306
Nerve fiber tortuosity	6.02 ± 2.66	5.61 ± 1.75	0.737

**Table 3:** Correlation *r* (*P* < 0.05) of corneal parameters in the T1DM group with clinical and metabolic data.

Corneal nerves parameters	Age	Age at onset	Duration of DM	BMI (kg/m^2^)	HBA1c (%)
Nerve fiber length (*µ*m/frame)	0.01	1.535 (0.018)^*∗*^	−0.657 (0.002)^*∗*^	−0.6 (0.007)^*∗*^	0.068
Nerve fiber length density (*µ*m/mm^2^)	−0.021	1.524 (0.021)^*∗*^	−0.666 (0.002)^*∗*^	−0.6 (0.007)^*∗*^	0.074
Number of fibers (no./mm^2^)	−0.060	1.444 (0.05)^*∗*^	−0.610 (0.006)^*∗*^	−0.587 (0.008)^*∗*^	−0.062
Number of branchings (no.)	−0.013	0.237	−0.305	−0.293	−0.210
Number of beadings (no.)	−0.228	0.088	−0.355	0.044	0.406
Beadings density (no./mm)	−0.210	−0.124	−0.075	0.404	0.292
Nerve fiber tortuosity	0.344	0.258	0.056	−0.202	−0.188

No significant correlations were found between the corneal parameters and HbA1c. BMI of the T1DM group was highly related to the nerve fiber length (*r* = −0.6, *P*=0.007), the nerve fiber length density (*r* = −0.6, *P*=0.007), and the number of fibers (*r* = −0.587, *P*=0.008) (Table 4).

**Table 4:** Comparison between the healthy control and T1DM patients divided by sex.

*Female*	Healthy control (*n* = 10)	T1DM (*n* = 10)	*P* value

Age (mean ± SD)	38 ± 4.3	39.4 ± 2.64	0.785
Nerve fiber length (*µ*m/frame)	979.44 ± 128.28	1018.09 ± 153.95	0.849
Nerve fiber length density (*µ*m/mm^2^)	11039.22 ± 1441.25	11456.11 ± 1732.28	0.855
Number of fibers (no./mm^2^)	5.70 ± 0.77	5.20 ± 0.61	0.619
Number of branchings (no.)	2.70 ± 0.54	2.00 ± 0.47	0.341
Number of beadings (no.)	18.4 ± 0.81	15.80 ± 2	0.244
Beadings density (no./mm)	68.87 ± 2.32	61.44 ± 7.3	0.345
Nerve nerve fiber tortuosity	5.16 ± 0.53	5.88 ± 0.50	0.336

*Male*	Healthy control (*n* = 9)	T1DM (*n* = 9)	*P* value
Age (mean ± SD)	42.89 ± 2.52	35.22 ± 3.21	0.079
Nerve fiber length (*µ*m/frame)	1415.42 ± 132.39	697.96 ± 101.48	0.001^*∗*^
Nerve fiber length density (*µ*m/mm^2^)	15932.77 ± 1489.73	7978.80 ± 1158.24	0.001^*∗*^
Number of fibers (no./mm^2^)	8.78 ± 0.83	4.11 ± 0.75	0.001^*∗*^
Number of branchings (no.)	3.89 ± 0.73	1.78 ± 0.57	0.026^*∗*^
Number of beadings (no.)	21.11 ± 1.36	16.67 ± 1.21	0.029^*∗*^
Beadings density (no./mm)	74.16 ± 4.23	70.27 ± 6.91	0.637
Nerve fiber tortuosity	6.98 ± 0.09	5.30 ± 0.65	0.070

**Table 5:** Comparison between male and female in the T1DM population.

	Male (*n* = 9)	Female (*n* = 10)	*P* value
Age (mean ± SD)	35.22 ± 3.21	39.40 ± 2.64	0.326
Age at onset of DM	19.55 ± 2.52	29.8 ± 3.19	0.045^*∗*^
Duration of DM (years)	15.67 ± 3.09	9.60 ± 2.05	0.113
BMI (kg/m^2^)	26.81 ± 1.75	23.42 ± 1.11	0.102
HBA1c (%)	7.59 ± 0.22	7.65 ± 0.32	0.880
TG (mg/dl)	90.55 ± 12.23	64.8 ± 5.56	0.063
CT (mg/dl)	170.66 ± 6.19	190.20 ± 9.72	0.117
HDL (mg/dl)	60.78 ± 5.28	71.40 ± 4.76	0.153
LDL (mg/dl)	91.78 ± 5.13	106.86 ± 9.57	0.197
Creatinine (mg/dl)	0.78 ± 0.04	0.76 ± 0.04	0.777
Microalbuminuria/creatininuria (mg/g)	7.6 ± 2.17	9.54 ± 3.04	0.617
Nerve fiber length (*µ*m/frame)	697.96 ± 101.48	1018.09 ± 153.95	0.05^*∗*^
Nerve fiber length density (*µ*m/mm^2^)	7978.80 ± 1158.24	11456.11 ± 1732.28	0.05^*∗*^
Number of fibers (no./mm^2^)	4.11 ± 0.75	5.20 ± 0.61	0.273
Number of branchings (no.)	1.78 ± 0.57	2.00 ± 0.47	0.735
Number of beadings (no.)	16.67 ± 1.21	15.80 ± 2	0.724
Beadings density (no./mm)	70.27 ± 6.91	61.44 ± 7.3	0.744
Nerve fiber tortuosity	5.30 ± 0.65	5.88 ± 0.50	0.253

**Table 6:** Correlation *r* (*P* < 0.05) of corneal parameters in the T1DM subgroup divided by sex and clinical data.

Corneal nerve parameters	Age		Age at onset		Duration of DM	
	Male	Female	Male	Female	Male	Female
Nerve fiber length (*µ*m/frame)	−0.347	0.051	0.403	0.441	−0.690 (0.040)^*∗*^	−0.620
Nerve fiber length density (*µ*m/mm^2^)	−0.398	0.051	0.372	0.441	−0.718 (0.029)^*∗*^	−0.620
Number of fibers (no./mm^2^)	−0.391	0.180	0.267	0.470	−0.625	−0.498
Number of branchings (no.)	−0.578	0.615	−0.105	0.503	−0.517	0.011
Number of beadings (no.)	−0.215	−0.233	0.121	0.170	−0.323	−0.564
Beadings density (no./mm)	−0.243	−0.103	−0.360	0.182	−0.040	−0.416
Nerve fiber tortuosity	0.605	−0.045	0.508	−0.038	0.216	0.001

**Table 7:** Correlation *r* (*P* < 0.05) of corneal parameters in the T1DM subgroup divided by sex and metabolic data.

Corneal nerve parameters	BMI		HBA1c	
	Male	Female	Male	Female
Nerve fiber length (*µ*m/frame)	−0.856 (0.003)^*∗*^	−0.358	−0.183	0.145
Nerve fiber length density (*µ*m/mm^2^)	−0.855 (0.003)^*∗*^	−0.358	−0.155	0.145
Number of fibers (no./mm^2^)	−0.774 (0.014)^*∗*^	−0.198	−0.426	0.159
Number of branchings (no.)	−0.579	0.154	−0.355	−0.133
Number of beadings (no.)	−0.094	0.097	0.040	0.535
Beadings density (no./mm)	0.452	0.276	−0.001	0.471
Nerve fiber tortuosity	−0.051	−0.316	0.201	−0.496

## Data Availability

The data used to support the findings of this study are available from the corresponding author upon request.
